# Whole-Genome Sequences of Streptococcus alactolyticus Strain An1F4 and Escherichia coli Strains Ae3A3 and Ae3B3, Isolated from Feces of Domestic Pigs

**DOI:** 10.1128/mra.00903-22

**Published:** 2023-01-10

**Authors:** Jens Jakob Sigurdarson, Satoshi Kawaichi, Simon Svane, Henrik Karring

**Affiliations:** a Department of Green Technology, University of Southern Denmark, Odense, Denmark; Wellesley College

## Abstract

Here, we present the genome sequences of a strain of Streptococcus alactolyticus and two strains of Escherichia coli that were isolated from feces samples from domestic pigs in Denmark. The genome sequences contribute to a better understanding of the microbiological processes in the feces and manure of domestic pigs.

## ANNOUNCEMENT

The production of domestic pigs (Sus domesticus) is associated with different environmental and societal challenges (1). The microbiological communities in pig feces and manure contribute to the production of different biogenic pollutants, including ammonia, methane, sulfur compounds, and volatile organic compounds (2). Hence, sequencing the genomes of bacteria isolated from pig feces will provide the basis for a better understanding of the microbiological and biochemical processes in pig feces and manure.

Streptococcus alactolyticus strain An1F4 and Escherichia coli strains Ae3A3 and Ae3B3 were isolated from feces samples from domestic pigs. Fresh pig feces samples were collected at Grønhøj Research Station (Karup, Denmark) without touching the pigs, and the animals were never stimulated or forced to excrete feces. Because the animals experienced no pain, suffering, anxiety, or lasting harm, approval from the Danish Inspectorate for Animal Experiments was not necessary, according to the relevant Danish legislation (Bekendtgørelse af lov om dyreforsøg). The feces samples were suspended in phosphate-buffered saline and used as an inoculum on a modified yeast casitone fatty acid (YCFA) agar medium without cysteine, sodium bicarbonate, and resazurin (3) and supplemented with 2 g/L glucose, 2 g/L maltose, and 2 g/L cellobiose as carbon and energy sources (4). Agar plates incubated under anoxic and oxic conditions at 37°C were used to isolate S. alactolyticus strain An1F4 and E. coli strains Ae3A3 and Ae3B3, respectively. The strains were purified by streaking single colonies onto new agar plates at least three times before the isolates were identified to the species level (identification scores of >2.3) using a matrix-assisted laser desorption ionization–time of flight mass spectrometry (MALDI-TOF MS) Biotyper system (Bruker Daltonics, USA). Single colonies of the purified S. alactolyticus and E. coli strains were grown in modified YCFA liquid medium at 37°C under anoxic and oxic conditions, respectively.

Genomic DNA of each strain was extracted from cultured cells using the standard protocol for the Gentra Puregene kit (Qiagen, Germany) before sequencing and processing by DNASense (Aalborg, Denmark). Paired-end sequence libraries (2 × 301 bp) were prepared using a NEBNext Ultra II DNA library preparation kit for Illumina (New England Biolabs, USA) and then were sequenced on a MiSeq instrument (Illumina, USA) using a MiSeq reagent kit v3 (600 cycles; Illumina, USA). Forward and reverse reads were trimmed based on adaptors and quality using Cutadapt v1.10 (5). The trimmed reads were assembled using SPAdes v3.7.1 (6) and mapped back to the respective assembled genomes (An1F4, Ae3A3, and Ae3B3) using BWA-MEM v0.7.17 (r1188) (7) to generate individual coverage profiles. Prodigal v2.6.3 (8), hmmsearch (HMMER v3.1b2) (9), and BLAST+ v2.7.1 (10) were used to find essential genes. MEGAN v4.70.4 (11) was subsequently used to extract consensus taxonomic assignments for the mmgenome coverage plots (12). Barrnap v0.8 (13) was used to extract the rRNA genes from each of the three assemblies (An1F4, Ae3A3, and Ae3B3), and the extracted 16S rRNA genes were classified using SINA v1.2.11 (https://www.arb-silva.de/aligner) (14). The results were analyzed in R v3.4.3 (15) through the RStudio integrated development environment (IDE) v1.1.383 using the mmgenome package v0.7.1 (12). The PhiX control DNA contig was removed from the assembly manually, and PhiX-screened genomes were annotated using the DDBJ Fast Annotation and Submission Tool (DFAST) v1.2.15 (16).

The genome of S. alactolyticus strain An1F4 has a total length of 1,699,682 bp, harbors 1,677 predicted coding sequences, and has a G+C content of 40%. E. coli strains Ae3A3 and Ae3B3 have genome sizes of approximately 5 Mbp, with 4,951 and 4,524 predicted coding sequences, respectively, and overall G+C contents of 51% (Table 1).

**TABLE 1:** Assembly results and statistics, numbers of essential genes, and accession numbers for the sequenced genomes of S. alactolyticus and E. coli strains isolated from pig feces

Bacterial isolate	Total length (bp)	No. of scaffolds	Size of longest scaffold (bp)	*N*_50_ (bp)	Mean scaffold length (bp)	Coverage (×)	Mean G+C content (%)	No. of predicted coding sequences	Total no. of essential genes	No. of unique essential genes	BioSample accession no.	GenBank genome accession no.
S. alactolyticus strain An1F4	1,699,682	75	128,731	32,139	22,662	157	40	1,677	107	106	SAMD00517187	BRXN01000000
E. coli strain Ae3A3	5,300,654	138	341,114	102,593	38,410	50	51	4,951	107	106	SAMD00517185	BRXL01000000
E. coli strain Ae3B3	4,874,471	159	193,214	70,132	30,657	54	51	4,524	107	106	SAMD00517186	BRXM01000000

### Data availability.

The assembly and annotation for the three genomes are available in DDBJ/ENA/GenBank. The genome accession numbers are BRXN01000000 for S. alactolyticus strain An1F4, BRXL01000000 for E. coli strain Ae3A3, and BRXM01000000 for E. coli strain Ae3B3.
